# Isolating auditory-nerve contributions to electrocochleography by high-pass filtering: A better biomarker for cochlear nerve degeneration?

**DOI:** 10.1121/10.0017328

**Published:** 2023-02-23

**Authors:** Viacheslav Vasilkov, M. Charles Liberman, Stéphane F. Maison

**Affiliations:** Eaton-Peabody Laboratories, Massachusetts Eye and Ear and Department of Otolaryngology —Head and Neck Surgery, Harvard Medical School, Boston, Massachussetts 02114, USA viacheslav.vasilkov@meei.harvard.edu; charles_liberman@meei.harvard.edu; stephane_maison@meei.harvard.edu

## Abstract

In search of biomarkers for cochlear neural degeneration (CND) in electrocochleography from humans with normal thresholds, we high-pass and low-pass filtered the responses to separate contributions of auditory-nerve action potentials (N_1_) from hair-cell summating potentials (SP). The new N_1_ measure is better correlated with performance on difficult word-recognition tasks used as a proxy for CND. Furthermore, the paradoxical correlation between larger SPs and worse word scores, observed with classic electrocochleographic analysis, disappears with the new metric. Classic SP is simultaneous with and opposite in phase to an early neural contribution, and filtering separates the sources to eliminate this interference.

## 1. Introduction

Studies of age-related hearing loss in animal models and human temporal bones have shown that cochlear nerve degeneration (CND) precedes hair cell loss (Sergeyenko *et al.*, 2013; Wu *et al.*, 2019). This neural loss does not elevate audiometric or electrophysiological thresholds until it becomes extreme (Woellner and Schuknecht, 1955; Chambers *et al.*, 2016), partly because the most vulnerable cochlear neurons do not contribute to threshold detection in quiet (Schmiedt *et al.*, 1996; Furman *et al.*, 2013). However, the silencing of these neurons degrades auditory processing and may compromise speech discrimination (Grant *et al.*, 2022), particularly in noisy environments (Monaghan *et al.*, 2020; Resnik and Polley, 2021; Wu *et al.*, 2021). Indeed, a number of studies have linked measures of speech perception or signal-in-noise detection with neural deficits assessed by auditory brainstem responses (ABRs)/electrocochleography (Bramhall *et al.*, 2015; Liberman *et al.*, 2016; Ridley *et al.*, 2018; Grant *et al.*, 2020; Lai and Bidelman, 2022), middle-ear muscle reflex (Mepani *et al.*, 2020; Shehorn *et al.*, 2020), envelope following responses (Mepani *et al.*, 2021; Marcher-Rorsted *et al.*, 2022), *in vivo* imaging of auditory nerve diameter (Harris *et al.*, 2021) or computational models (Buran *et al.*, 2022). Furthermore, CND and the loss of afferent activity it produces may trigger an enhancement of central gain that further degrades performance on complex listening tasks (Oxenham, 2016; Parthasarathy *et al.*, 2020; Resnik and Polley, 2021).

In animal studies, CND can be directly measured by counting synapses between inner hair cells and auditory nerve fibers (ANFs). Loss of synapses is highly correlated with the reduction of suprathreshold amplitudes of ABR wave 1, so long as cochlear thresholds remain normal (Kujawa and Liberman, 2009). A typical ABR response to high-level clicks, alternated in polarity to remove hair-cell microphonic potentials, includes a prominent negative peak at around 1 msec, called N_1_ (or AP or wave 1), and an inflection on its rising phase called the SP. N_1_ is dominated by action potentials (spikes) of ANFs, while the SP includes contributions from hair cell receptor potentials and ANF post-synaptic potentials (Durrant *et al.*, 1998; Pappa *et al.*, 2019; Lutz *et al.*, 2022).

Inferring CND from ABRs in humans is more challenging because the recordings are noisy, and N_1_ is small when using conventional electrodes and montage. Measuring responses with electrodes in the ear canal or directly on the eardrum, i.e., electrocochleography (EcochG), increases response amplitudes. However, even with intra-meatal electrodes, N_1_ amplitudes remain highly variable across subjects, even among those with normal audiograms (Grant *et al.*, 2020).

We hypothesize that N_1_ amplitude variability, at least in part, may be related to CND, i.e., the peripheral neural deficit that cannot be explained by a loss of outer hair cells. To pursue this idea, we and others have looked for correlations between the variability of N_1_ responses and performance on a variety of difficult word-recognition tasks in normal-threshold subjects as a proxy for CND. Some have found correlations consistent with the contribution of CND to intelligibility (Bramhall *et al.*, 2015; Grant *et al.*, 2020; Mepani *et al.*, 2020; Lai and Bidelman, 2022), and others have not (Prendergast *et al.*, 2017; Guest *et al.*, 2018). Some of the discrepant outcomes may arise because of differences in the evoked-response metrics: e.g., baseline to N_1_ peak (Liberman *et al.*, 2016), N_1_ peak to P_1_ trough (Prendergast *et al.*, 2017; Bramhall *et al.*, 2019; Couth *et al.*, 2020), or SP peak to N_1_ peak (Grant *et al.*, 2020; Mepani *et al.*, 2020), and/or from differences in the methods for data acquisition (including filter bandwidths) or extraction, i.e., visual inspection (Prendergast *et al.*, 2017; Grant *et al.*, 2020) or mathematical modeling (Valderrama *et al.*, 2014; Kamerer *et al.*, 2020; Hancock *et al.*, 2021).

A paradoxical result from prior studies of CND biomarkers in humans has been the observation that SP-related metrics are correlated with performance on word-in-noise recognition tests, wherein SP amplitude increases as performance declines (Liberman *et al.*, 2016; Grant *et al.*, 2020; Lai and Bidelman, 2022). In animal studies of synaptopathy, SP amplitude is unchanged as N_1_ decreases (Kujawa and Liberman, 2009; Sergeyenko *et al.*, 2013), as expected if ANFs are silenced without permanent hair cell damage, and if SP is dominated by hair cell receptor potentials. Although it must be noted that ABR responses in these animal studies were filtered through a 300–3000 Hz passband that removes most of the SP energy: see Fig. 2 from Hancock *et al.* (2021).

Although the analysis of evoked response waveforms in the time domain provides important cues regarding the generators that evoke them, contributions of ANF spikes cannot be cleanly separated from hair cell or ANF post-synaptic responses, as they can overlap in time (Pappa *et al.*, 2019; Lutz *et al.*, 2022). Here, in hopes of identifying cleaner biomarkers of CND in humans, we try to improve the separation of ANF responses from the SP by filtering the EcochG waveforms into a high-pass and a low-pass component, with a cutoff near 500 Hz to isolate the 800 Hz spectral peak attributed to contributions of ANF spikes. This 800 Hz neural peak dominates the spectrum of the electrical noise recorded at the round window (in quiet) and disappears when ANF spikes are pharmacologically blocked (Dolan *et al.*, 1990). Similarly, sound-evoked EcochGs show a spectral peak near 800 Hz (Hancock *et al.*, 2021), which is absent in patients with otoferlin mutations that disrupt transmitter release from the inner hair cell synapses (Santarelli *et al.*, 2019; Hancock *et al.*, 2021), consistent with its association with ANF spikes. Furthermore, the single-neuron contribution to a gross potential derived by cross-correlating the spontaneous spike trains of single ANFs with the round-window electrical noise has a periodicity of ∼1.25 ms, which produces the spectral peak near 800 Hz (Kiang *et al.*, 1976; Prijs, 1986).

This high-pass filtering of the EcochG waveform enhances the correlations between word scores and a metric of ANF activity. It also explains the paradoxical increase in SP amplitude among the worst performers because the high-pass filtered (neural spiking) component has an initial negative phase coincident with the SP, such that reducing the ANF spiking component (as in synaptopathy) must increase SP amplitude as it decreases N_1_.

## 2. Materials and methods

### 2.1 Subject pool, cognitive assessment, and inclusion criteria

122 native speakers of English, in good health, between the ages of 18 and 63, with no history of ear or hearing problems, no history of neurologic disorders, and unremarkable otoscopic examinations were recruited. All participants had normal audiometric thresholds from 250 Hz to 8 kHz in both ears and normal middle-ear function. A thorough description of behavioral threshold assessments (standard and extended high frequencies) and tympanometric measures for most of the same subjects has been described in prior reports (Grant *et al.*, 2020; Mepani *et al.*, 2020). All participants included in this study passed the Montreal Cognitive Assessment (scores ≥ 26) that screens for mild cognitive dysfunction. There were no additional inclusion criteria beyond the ability to give voluntary informed written consent. This study was reviewed and approved by the Institutional Review Board of the Massachusetts Eye and Ear.

### 2.2 Word recognition

Word-recognition performance was assessed by counting the number of correctly repeated words from a list of 50 phonemically balanced words from the Northwestern University Auditory Test No. 6 (NU-6) corpus presented at 55 dB hearing level (HL) (∼75 dB sound pressure level, SPL) with “time compression” and added reverberation (65% reduced duration with 0.3 s echo) (Noffsinger *et al.*, 1994). This NU-6 test will be subsequently referred to as the “65%” test. We also used a modified version of the QuickSIN™ (mQSIN) Speech-in-Noise test (Etymotic Research, Inc., Elk Grove Village, IL) consisting of four lists of six sentences with five key words per sentence in the presence of a four-talker babble noise at decreasing SNR from 10 to 5, 3, 2, 1, and 0 dB (see Mepani *et al.*, 2020). The first list of six sentences was used as practice. A combined score for the three subsequent lists consisted of adding the number of correctly repeated key words.

### 2.3 Electrocochleography

Stimuli were generated by a custom rig and transduced *via* ER-3A insert earphones, and data acquisition was handled by the Interacoustics Eclipse hardware and software. While trans-tympanic needle electrodes or tympanic membrane electrodes provide larger electrophysiological responses, we favored the use of ear canal electrodes (tiptrodes) to provide better comfort to our participant. Subjects' ear canals were prepped by scrubbing with a cotton swab coated in Nuprep^®^ Electrode gel (Nuprep, Aurora, CO) was applied on the cleaned portion of the canal and over the gold-foil of ER3-26 A/B tiptrodes before insertion. A horizontal montage was used, with a ground on the forehead at midline, one tiptrode as the inverting electrode, and the other as the non-inverting electrode in the opposite ear. Low (<5 kΩ) and balanced impedance readings were obtained with inter-electrode impedance values within 2 kΩ of each other. Acoustic stimuli were delivered *via* silicone tubing connected to the ER-3A earphones. Stimuli were 100 *μ*s-clicks delivered at 125 dB peak SPL in alternating polarity at a presentation rate of 9.1 or 40.1 Hz. The total noise dose for all EcochG measurements was well within Occupational Safety and Health Administration (OSHA) and National Institute for Occupational Safety and Health (NIOSH) standards. Electrical responses were amplified 100 000 times, and 2000 sweeps were averaged for each recording.

Average traces acquired by the Eclipse software (passband 3.3 Hz–5000 Hz) were exported to matlab R2018a for further analyses using custom scripts. Specifically, EcochG waveforms were processed using standard *highpass()*/*lowpass()* matlab functions with infinite impulse “*iir”* response type, a stop band attenuation of 60 dB and a “*steepness”* argument of 0.95 (resulting in a filter slope of 38.8 dB/octave). The cutoff frequencies were 3.3–470 Hz for the low-pass filter and 470–3000 Hz for the high-pass filter.

### 2.4 Statistical analysis

A paired Student's *t* test was used to assess differences within each group under different conditions. Pairwise Pearson's correlations were used to assess the relationships between EcochG metrics and word recognition scores. A two-tailed Student's *t* test for homoscedastic groups was used to test for a difference in the mean EcochG metrics between the best and worst performers of each word recognition test (below 25th and above 75th percentile). The threshold for statistical significance was *p* = 0.05.

## 3. Extracting the neural spiking components from EcochG responses

**Fig. 1.**
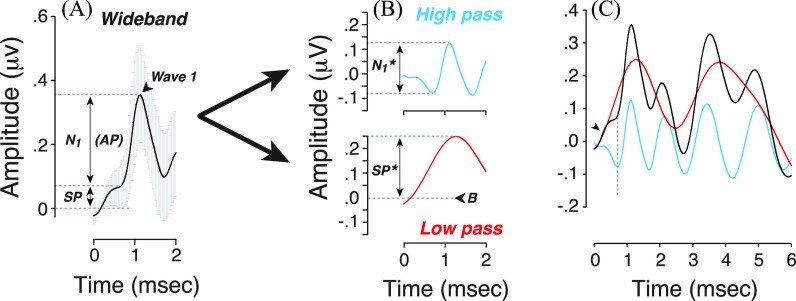
(A) Averaged click-evoked EcochG obtained from all participants (+/− standard deviation, SD). Baseline (B) was defined as the first amplitude point > 2 SD above the mean pre-onset amplitude (−2 to 0 ms). When extracted by visual inspection, the SP is defined as the difference between baseline and the last inflection point on the rising phase of the first waveform peak; N_1_ is defined as the amplitude difference between SP and the first peak (1–2 ms after stimulus onset). (B) A low-pass (3.3–470 Hz; red) and high-pass (470– 3000 Hz; blue) filtered version of the mean wideband waveform from (A). SP^*^ is defined as the baseline-to-peak amplitude of the first wave on the low-passed waveform; N_1_^*^ is defined as the trough-to-peak amplitude of the first wave on the high-passed waveform. (B) indicates the latency at which baseline is measured. (C) Superimposed waveforms show the overlap of the low-pass component with the wideband (original) waveform in the first 0.5 ms, where SP dominates the response (arrowhead). The dotted line indicates the latency of the inflection point where N1 rises from the SP.

To separate the contributions of ANF spikes from other generators, we first processed EcochG waveforms using a Fast Fourier Transform (FFT) with a −2 to 8 ms time window without subtracting the mean of the response, and without applying a hamming window function to preserve the original signal (in contrast to what was performed in Hancock *et al.* (2021). In addition, each waveform was zero-padded to increase the frequency resolution of the FFT to 3.66 Hz. The first spectral trough after the 300 Hz peak (see Hancock *et al.*, 2021) was selected as the filter cut-off frequency (470 Hz). Each EcochG waveform was then analyzed after high-pass and low-pass filtering (Fig. 1). The selected cutoff frequency provided low-pass filtered waveforms that match the rising slope of the SP in the unfiltered waveform [Fig. 1(C)]; however, the exact position of the cutoff can be varied between 300 and 600 Hz with only minimal changes in the subsequent results.

On the premise that our high-pass filtered waveform is dominated by neural spikes, we define the trough-to-peak amplitude within the first 1.5 ms, i.e., N_1_^*^, as a new and objective measure of ensemble ANF response [Fig. 1(B)]. Given that the low-pass EcochG waveform is monotonically rising for latencies < 1 ms [Fig. 1(B)], we have arbitrarily chosen to measure SP^*^ amplitude as the baseline-to-peak amplitude. As in prior studies, the baseline was defined at the first point exceeding two standard deviations above the mean pre-onset waveform amplitude (−2 to 0 ms) prior to filtering [Fig. 1(A), *wideband*]. For each filtered waveform, baseline is defined as the amplitude measured at the same latency.

## 4. Reinterpreting the association between sp amplitude and word scores

Extracting the putative ANF spiking component from other sources, including hair cell receptor potentials and non-spiking neural components, revealed two related points. First, the morphology of the high-pass waveform within the first 2 ms is triphasic, as can be seen for extracellular potentials from spiking activity (Johnstone and Wu, 1995; Barry, 2015). A triphasic morphology arises when the nodes of Ranvier dominating the response alternate from current sources to sinks and back to sources as the action potential propagates from upstream to downstream of the nodes in question, which in electrocochleography may be at ANF cell bodies in the spiral ganglion. Although a biphasic waveform for the contribution of ANF spiking to round-window potentials has been inferred in normal gerbils by subtracting responses measured after kainate blockade from the pre-kainate potentials (Pappa *et al.*, 2019; Lutz *et al.*, 2022). It is likely that many of the humans in our study also have basal-turn hair cell lesions, given the wide range of thresholds seen at extended high frequencies (Grant *et al.*, 2020).

**Fig. 2.**
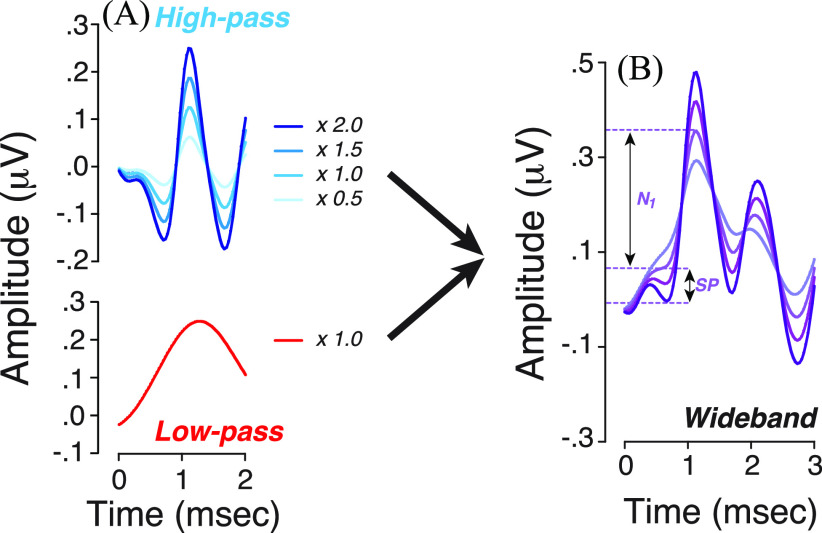
SP amplitude, as classically measured, increases as the putative ANF spiking component (high-pass) decreases. (A) The high-pass filtered waveform was reweighted from half to twice its original value (as indicated in key) and added back to a constant the low-pass component. (B) Outcomes of changes produced in (A) on EcochG waveforms.

Second, the opposing phases of the high- and low-pass components within the first ms of the response [Fig. 1(B)] suggest that SP amplitude, as classically measured, will increase as the ANF contributions decrease. To show quantitatively the dependence of SP amplitude on the putative neural spiking component, we re-weighted the high-pass filtered waveform from half to twice its original value [Fig. 2(A)] and added it back to a constant low-pass component [Fig. 2(B)]. This analysis provides a new way to think about the paradoxical result from prior studies of CND biomarkers, which found that SP amplitude was correlated with speech-in-noise performance, with the largest SPs among those with the worst scores (Liberman *et al.*, 2016; Ridley *et al.*, 2018; Grant *et al.*, 2020; Mepani *et al.*, 2020). We conclude that a rise in SP, as classically measured, may reflect a loss of ANFs rather than a set of complicated interactions between inner and outer hair cell contributions and post-synaptic currents from ANF terminals as we previously speculated (Liberman *et al.*, 2016; Grant *et al.*, 2020; Hancock *et al.*, 2021).

**Fig. 3.**
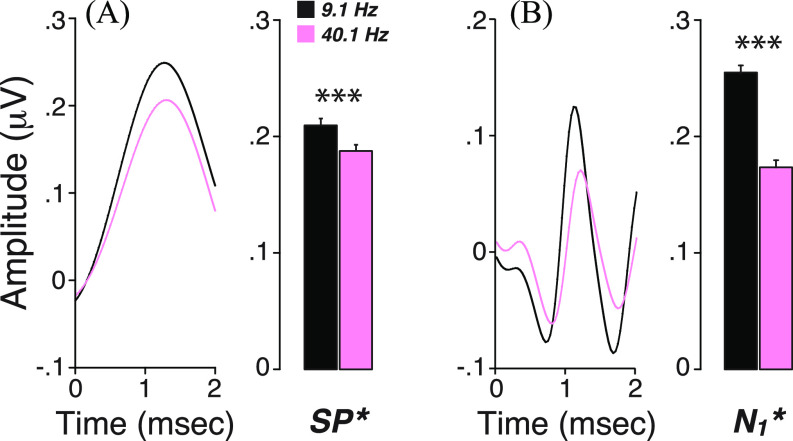
Both low- and high-pass waveforms are attenuated by increasing click rate. Mean low-pass (A) vs high-pass (B) EcochG waveforms and their respective metrics (mean ± SEM, standard error of the mean) are compared for two click repetition rates (9.1 vs 40.1 Hz). ^***^p < 0.001. SP^*^ and N_1_^*^ are measured as shown in Fig. 1.

To further assess the contributions of ANFs to the high-pass components and gain insight into the cellular generators of the low-pass component, we compared EcochG waveforms acquired at different click rates (40.1 Hz vs 9.1 Hz), because hair cell potentials are not attenuated by high-repetition rates (Kiang and Peake, 1960), whereas neural potentials should show strong adaptation (Eggermont and Odenthal, 1974). As shown in Fig. 3, both the high-pass and low-pass amplitudes decreased significantly with an increased presentation rate (*p* < 0.001 for both SP^*^ and N_1_^*^). While this result was expected for the former, the attenuation of the latter suggests a contribution of non-spiking neural components, possibly in the form of post-synaptic potentials. This result is consistent with our previous study showing a correlation between one component of SP^*^ (the spectral magnitude of EcochG near 300 Hz) with both SP and N_1_ amplitudes (Hancock *et al.*, 2021) and with animal studies suggesting that SP has a neural component in addition to a hair cell component (Pappa *et al.*, 2019; Lutz *et al.*, 2022).

## 5. Use as a biomarker of cochlear nerve degeneration

**Fig. 4.**
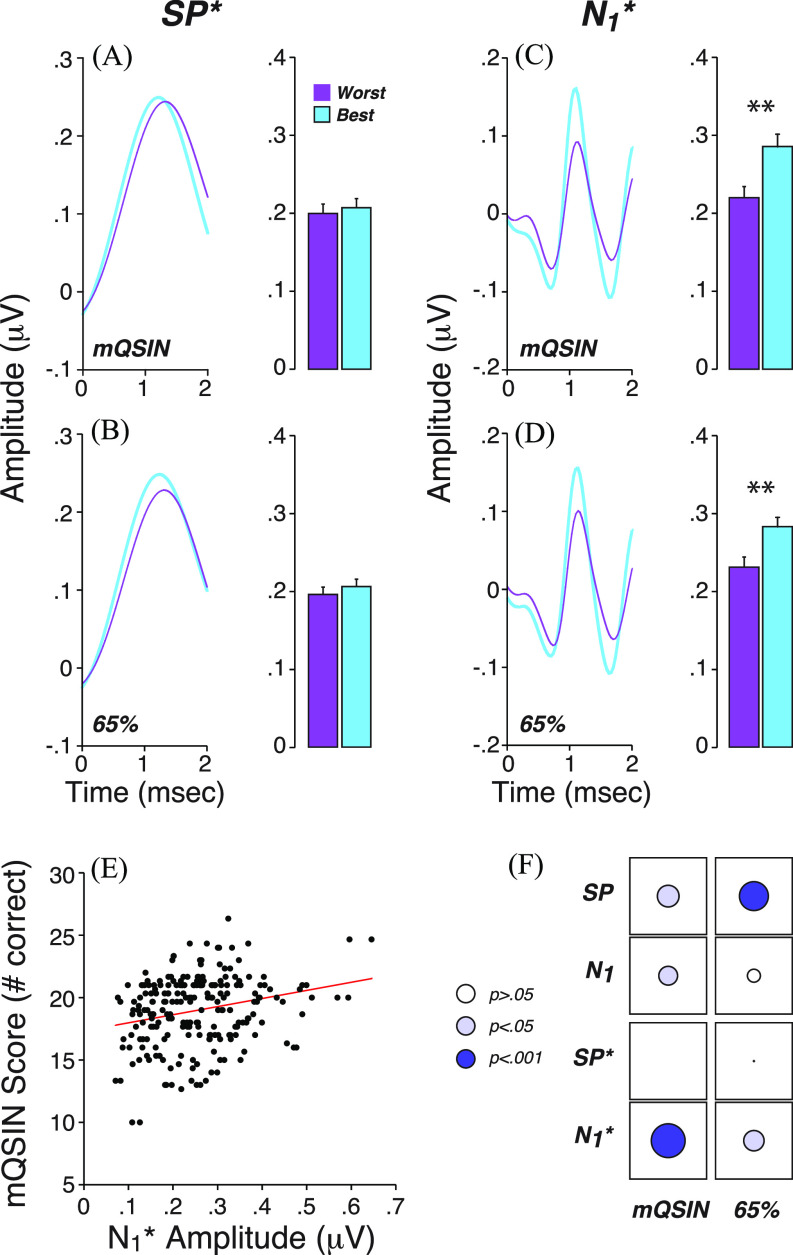
Performance on word recognition tests is correlated with N_1_^*^, and not with SP^*^. (A)–(D): Mean filtered EcochG waveforms and their respective metrics (mean ± SEM) for the best (blue) vs worst (purple) performers on word tests: modified QuickSIN (mQSIN) or 65% time compression with reverberation (65%). Best and worst performers represent the top and bottom quartiles of the participants. (A) and (B) vs (C) and (D) show mean data for low-pass vs high-pass waveforms. ^**^p < 0.01. (E) Illustrates the correlation between scores on the modified QuickSIN and N_1_^*^ amplitude. (F) Correlation matrix between word scores and unfiltered (top) vs filtered (bottom) EcochG metrics. The diameter of each disk is proportional to the *r* value of Pearson's bivariate correlations. The disk color indicates the level of statistical significance as indicated in key. SP^*^ and N_1_^*^ are measured as shown in Fig. 1.

CND has been implicated in the intelligibility challenges of “normal” hearing or hearing-impaired subjects, especially in difficult listening situations. If the filtering approach described here extracts the neural spiking component from EcochG waveforms, it might provide a cleaner biomarker of CND in humans. To evaluate this, we compared the correlations between word-recognition scores, our proxy for CND, and the new vs old metrics of N_1_ and SP extracted from EcochG waveforms (Fig. 4).

As previously described (Mepani *et al.*, 2020), the use of difficult word recognition tests that include “time compression” and added reverberation or a competing babble background at difficult SNRs (mQSIN) spreads the scores obtained from “normal” hearing participants: the 65% compression with reverberation yields many scores in the 20%–60% range. A similar range of scores was observed with a modified version of the QuickSIN with scores ranging from 10 to 26 correctly repeated key words out of 30.

We first compared participants who scored best vs worst on the word tests, i.e., with scores above the 75th and below the 25th percentile, respectively. Mean SP^*^ was similar between best and worst performers with no significant difference for either the modified QuickSIN test [*p* > 0.05, Fig. 4(A)] or for words presented with 65% time compression and reverberation [*p* > 0.05, Fig. 4(B)]. In contrast, significantly larger N_1_^*^ amplitudes were seen for the best performers on either word test [mQSIN: *p* = 0.003, Fig. 4(C); 65%: *p* = 0.004, Fig. 4(D)].

When assessing data from all participants [Figs. 4(E) and 4(F)], the correlations between word scores and N_1_^*^ amplitude were significant for both word tests (mQSIN vs N_1_^*^: *r* = 0.24, *p* < 0.001; 65% vs N_1_^*^: *r* = 0.15, *p* = 0.021), and the significance levels were higher than when “neural” metrics were extracted in the classic way [mQSIN vs N_1_: *r* = 0.16, *p* < 0.05; 65% vs N_1_: *r* = 0.10, *p* = 0.12, Fig. 4(F)]. Similarly, when using the classic metrics, SP was correlated with word scores, in the paradoxical direction that larger SPs were associated with worse word scores (*p* values for “SP” in Fig. 4(F)]. When extracted from the low-pass filtered waveforms, the correlations between the “summating potential” and word scores disappeared (mQSIN: *r* = −0.007, *p* > 0.05; 65%: *r* = −0.016, *p* > 0.05) [Fig. 4(F)].

## 6. Conclusion

This study offers a new approach to EcochG analysis that **(**1) may more cleanly separate the neural spiking component from other cellular generators and **(**2) can be carried out objectively under computer control. While further animal studies are needed to confirm that the neural spiking component is effectively separated from other generators, we believe that this approach may be useful in the ongoing search for CND biomarkers in humans. Future analyses looking at differences in responses obtained from rarefaction vs condensation clicks may further clarify the generators underlying these responses.

Consistent with a role for CND in speech intelligibility deficits, especially in difficult listening environments, we found correlations between word scores and the first peak of the EcochG after high-pass filtering above 470 Hz, which were stronger than those seen when N_1_ amplitudes were measured in the conventional way. This filtering approach also suggests that the earliest contribution of ANF spiking to the EcochG overlaps in time with, and is opposite in phase, to the SP as conventionally measured. Thus, a conventional SP will rise in amplitude as the ANF contributions decrease in magnitude, which may explain the strong association between higher SP amplitudes and lower word scores observed in prior studies of CND biomarkers.
